# Inertial Sensor-Based Step Length Estimation Model by Means of Principal Component Analysis

**DOI:** 10.3390/s21103527

**Published:** 2021-05-19

**Authors:** Melanija Vezočnik, Roman Kamnik, Matjaz B. Juric

**Affiliations:** 1 Faculty of Computer and Information Science, University of Ljubljana, Večna Pot 113, 1000 Ljubljana, Slovenia; matjaz.juric@fri.uni-lj.si; 2 Faculty of Electrical Engineering, University of Ljubljana, Tržaška Cesta 25, 1000 Ljubljana, Slovenia; roman.kamnik@fe.uni-lj.si

**Keywords:** gait model, inertial sensors, open-source dataset, smartphone, step length estimation model

## Abstract

Inertial sensor-based step length estimation has become increasingly important with the emergence of pedestrian-dead-reckoning-based (PDR-based) indoor positioning. So far, many refined step length estimation models have been proposed to overcome the inaccuracy in estimating distance walked. Both the kinematics associated with the human body during walking and actual step lengths are rarely used in their derivation. Our paper presents a new step length estimation model that utilizes acceleration magnitude. To the best of our knowledge, we are the first to employ principal component analysis (PCA) to characterize the experimental data for the derivation of the model. These data were collected from anatomical landmarks on the human body during walking using a highly accurate optical measurement system. We evaluated the performance of the proposed model for four typical smartphone positions for long-term human walking and obtained promising results: the proposed model outperformed all acceleration-based models selected for the comparison producing an overall mean absolute stride length estimation error of 6.44 cm. The proposed model was also least affected by walking speed and smartphone position among acceleration-based models and is unaffected by smartphone orientation. Therefore, the proposed model can be used in the PDR-based indoor positioning with an important advantage that no special care regarding orientation is needed in attaching the smartphone to a particular body segment. All the sensory data acquired by smartphones that we utilized for evaluation are publicly available and include more than 10 h of walking measurements.

## 1. Introduction

Over the past few decades, gradual advances in the development of microelectromechanical systems (MEMS) technology have laid the foundations for bulk inertial sensors production and the subsequent penetration of those sensors to the market [1]. Their demand is currently not only predominant in the Internet-of-Things (IoT) sector but is extending to Industry 4.0 as well [2]. A vast array of promising applications is readily emerging for location-based services and healthcare due to the integration of MEMS-based inertial sensors into IoT wearables and smartphones. So far, authors have studied the gait of patients with certain diseases or conditions that impact the walking pattern, e.g., stroke [3,4], Parkinson’s disease [5,6,7,8], or Huntington’s disease [9,10]. Other such studies monitor a person’s activity and movement in urban areas using approaches, such as activity recognition [11,12,13], gait authentication [14,15,16], and PDR-based indoor positioning [17,18,19,20,21,22].

PDR-based approaches calculate current position based on the change in the previous position by using the information on step length and heading. Proposed techniques for step length estimation vary in terms of the implementing means of deriving step length. For example, machine learning techniques are often utilized in the derivation process in combination with various statistical estimation methods to improve the estimation accuracy, i.e., Bayesian filters such as the Kalman filter [23,24], the particle filter [25,26,27], and grid-based approaches [28]. Similarly, artificial neural networks [29] are also known to be employed. The aforementioned techniques often require extensive training prior to utilization. Step length is calculated by integrating the acceleration in walking direction over time as well [8,30,31]. It usually requires the elimination of drift during the calculation process and the placement of sensors on the shank, on the foot, or on the center of body mass to achieve the optimal step length estimation accuracy. Moreover, step length is also estimated by a model [18,19,20,21], often as the linear combination of inertial sensor outputs, such as step frequency or acceleration. Due to its applicability, this approach is most commonly used for smartphone-based step length estimation in PDR-based approaches [32]. This is the main reasoning for the idea presented herein.

Some step length estimation models exploit the direct empirical correlation between inertial sensor outputs and step length by including tunable constants. Of note, Weinberg [33] based the model on the difference between the maximum and minimum vertical acceleration values within the step while Kim et al. [34] based their model on mean absolute acceleration value in walking direction within the step. Alvarez et al. [35] exploited the linear relationship between step frequency and step length. Similar to Alvarez et al. [35], Renaudin et al. [36] and Zhang et al. [37] also exploited the linear relationship between step frequency and step length in their models, whilst also incorporating the user’s height (Renaudin et al. [36]) and leg length (Zhang et al. [37]). Tian et al. [21] based their model on the square root of step frequency and user’s height, whereas Sharp and Yu [38] based their model on the user’s height, step frequency, and the difference between maximum in minimum vertical acceleration values within the step. Shin and Park [39] proposed a model that estimates step length as the linear combination of step frequency and acceleration variance within the step. Similarly, Sun et al. [40] based their model on the variance of vertical acceleration and step frequency.

Another basis for the derivation of the models could potentially utilize physical models of gait. For example, the inverted pendulum model [41] could be used for determining the displacement of the pelvis during walking. Zijlstra and Hof [42] used vertical displacement of the center of body mass within the step and user’s leg length as the basis of the model and included one tunable constant. Do et al. [19] and Lan and Shih [43] used the same basis as Zijlstra and Hof [42] in their models, and they did not include any tunable constants. On the contrary, Diaz and Gonzales [44] exploited the linear relationship between the step length and the maximum angle between legs within the step by including two tunable constants in the model. Some authors also extended existing models to improve the accuracy of step length estimation. As such, Mikov et al. [45] extended the Weinberg model [33] with the inverse of step frequency, whereas Kang and Han [20] extended the Weinberg model [33] with an additional tunable constant and included another equation, in which they replaced the fourth principal root with a logarithm. Zhu et al. [46] extended the Weinberg model [33] with the acceleration variance and step frequency. Guo et al. [47] extended the model proposed by Kim et al. [34] with a tunable constant, whereas Bylemans et al. [18] extended the model proposed by Kim et al. [34] with step frequency and the difference between the maximum and minimum vertical acceleration values within the step.

Even though certain models performed well on the datasets prepared according to the specific evaluation protocol, we have already demonstrated that their performances change under different evaluation protocols [32]. Certain models are affected by smartphone position or walking speed, acceleration-based models in particular, yet they are well established for step length estimation in PDR-based approaches. However, kinematics associated with different body segments during walking in combination with measured step lengths are rarely used in the derivation processes of these models. By considering the aforementioned reasons, we designed a study for the derivation and evaluation of an acceleration-based step length estimation model using an optical measurement system and several off-the-shelf smartphones. Firstly, we aimed to investigate the kinematics of the human body during walking to identify key characteristics of the movement of anatomical landmarks. Gaining insights into their similarities would enable us to derive a step length estimation model, suitable for various smartphone positions, such as in a pocket, in a hand, or on the upper arm. In the derivation process, we, therefore, utilized PCA that provides enhanced insight into data by reducing the dimensionality of the dataset whilst retaining a large degree of variation from the original dataset [48,49,50]. To the best of our knowledge, we were the first to utilize PCA to derive a step length estimation model. So far, PCA has been successfully employed in gait analysis to support the kinematic design of the wearable walking assistive device for use by hemiplegics [51]. PCA was also used to examine movement behavior in children [52] identifying key facets of human movement. For example, Hinkel-Lipsker and Hahn [53] employed PCA to reduce the number of dimensions in studying gait kinematics.

The next aim of the work presented herein was to evaluate the proposed model on smartphones for different walking speeds and typical positions. We used four off-the-shelf smartphones in the evaluation reproducing scenarios of typical smartphone users while using an optical measurement system to track the smartphones’ spatial positions. Therefore, smartphones were attached to the upper arm, the pelvis, the hand, and to the thigh to simulate typical scenarios, e.g., putting a smartphone in a sports bag attached to the upper arm or pelvis while walking or carrying the smartphone in a hand or pocket while walking.

We propose a new step length estimation model that utilizes acceleration magnitude as the main input. This is an important advantage over the compared acceleration-based models that no special care regarding orientation is needed when attaching the smartphone to a particular body segment. We evaluated the performance of our proposed model for four typical smartphone positions on the user’s body for several walking speeds during long-term walking. We obtained promising results: the proposed model outperformed the compared acceleration-based models in terms of the overall accuracy of estimated walked distance, with the mean absolute stride length error being 6.44 cm. Furthermore, our proposed model was also least affected by walking speed and smartphone position among acceleration-based models.

The rest of the paper is structured as follows. Section 2 presents the derivation of the model where the design of the study, evaluation protocol, data analysis, the formulation of our proposed model by means of PCA, and the evaluation are described. Section 3 presents the results, while Section 4 discusses them. Finally, Section 5 concludes the paper.

## 2. Methods

The formulation of a step length estimation model follows an established approach consisting of a study for deriving the model and experiments for evaluating it. The study usually includes experiments to acquire sensor data from a particular position on a person’s body and the analysis of these data exploiting the correlation between step length and certain inertial sensor outputs. The formulated model is, thereafter, evaluated on another, larger dataset that includes the sensor data acquired in the experiments conducted under different circumstances, where usually walking speed, the duration, terrain, or sensor position vary in each trial.

Instead of adopting the described standard approach for the derivation of the model, we shifted towards a more unconventional one. We did not exploit the direct correlation between step length and certain inertial sensor outputs but used the reference data collected from anatomical landmarks on the human body instead. After the initial data acquisition, we employed PCA to identify patterns and the correlations between the walked distance and the minimum number of input parameters [48,49,50]. Based on our findings, we proposed a new step length estimation model and evaluated it on another dataset.

### 2.1. Design of the Study

**Figure 1:**
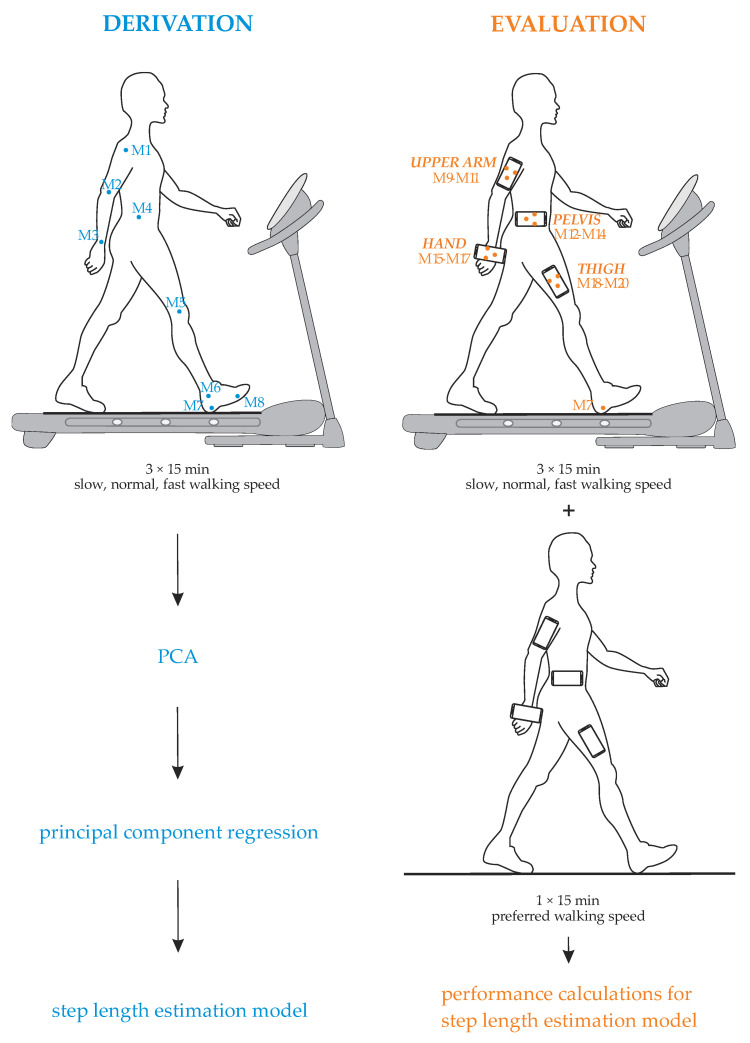
Design of the study for the derivation and evaluation of the step length estimation model.

This work aimed to derive and evaluate a new acceleration-based step length estimation model. Herein, we planned an outline of our study as shown in Figure 1. The first part was focused on the derivation of the step length estimation model, whereas the second part was dedicated to the evaluation of the proposed model.

Firstly, we studied the motion of the human body during long-term walking for several walking speeds and its impact on the walked distance when walking on a treadmill. The motion on one side of the human body was investigated, since the walking of a non-elderly healthy person can be considered symmetrical regardless of gender [54]. Therefore, walking was analyzed on the level of gait cycles of one limb. A gait cycle is the interval between successive foot contacts on the same limb [55]. Stride lengths were measured for each gait cycle and used in the study. The positions of anatomical landmarks on the human body were measured during slow, normal, and fast walking using the optical measurement system Optotrak Certus (Northern Digital Inc., Waterloo, ON, Canada). The Optotrak system tracked the spatial positions of infrared markers Mi = (x_i_(t), y_i_(t), z_i_(t)), 1 ≤ i ≤ 8, with an accuracy up to 0.1 mm. The markers were attached to anatomical points of the shoulder (acromion process), elbow (lateral epicondyles), wrist (ulnar styloid process), between hip (greater trochanter) and pelvis (upper iliac crest), knee (lateral femoral condyle), ankle (lateral malleolus), heel (lateral process of calcaneal tubercle), and toes (metatarsophalangeal joint). The acquired data were characterized by employing PCA. This enabled the identification of relevant parameters common to all anatomical landmarks that met the following criteria: on one hand, they had the greatest impact on the motion of the human body during walking, but on the other hand, they could be used as a basis for a light-weight accurate acceleration-based solution for step length estimation, suitable for smartphones. Applying these criteria would, therefore, result in proposing a step length estimation model that requires the minimum number of input parameters to minimize the pre-processing while including one of the acceleration-based parameters that vary the most within the step as the basis. After, we employed principal component regression to obtain the correlation between stride length and certain parameters, which formed a new model.

In the second part of our work, we evaluated the proposed model with the selected performance metrics for two types of walking modes: (i) walking on a treadmill and (ii) walking on a rectangular-shaped test polygon. Both walking modes were evaluated for long-term human walking with a range of different walking speeds and four smartphone positions, i.e., smartphone attached to the upper arm, hand, pelvis, and thigh. These positions were chosen as we tried to reproduce scenarios of typical smartphone users while using the optical measurement system to track the spatial positions of smartphones. The performance of the proposed model was compared to the performances of the related acceleration-based models. The data used in the evaluation are part of our open benchmark repository [56].

### 2.2. Experimental Protocol

Different walking speeds were tested on the treadmill for the derivation and evaluation of the step length estimation model, the values of which were determined as the average values of self-selected persons’ slow, normal, and fast walking speeds from our previous work [32]. As a result, 3.3, 4.6, and 5.9 km/h were tested for slow, normal, and fast walking speeds, respectively. An additional set of experiments were conducted on a 21.64-meter-long rectangular-shaped test polygon where the person self-selected their walking speed. They were asked to choose their preferred walking speed and to maintain it while walking. Both sets of experiments lasted for approximately 15 min for each walking speed. Altogether 10 individuals (six men and four women) participated in the experiment, all selected from a group of healthy adults aged from 19 to 32 years (mean value of 26.3 ± 4.6 years) at the time of the experiment. Their height ranged from 1.60 to 1.83 m (mean value of 1.77 ± 0.10 m), whereas their leg length varied from 0.90 to 1.14 m (mean value of 1.05 ± 0.07 m).

Four off-the-shelf smartphones (Samsung S8, Samsung S7 edge, and two Samsung S2) were used as shown in Figure 1 to acquire linear acceleration sensor data on the upper arm, hand, pelvis, and thigh. On the treadmill, linear accelerations were measured along with positions of 12 infrared markers placed on smartphones Mi = (x_i_(t), y_i_(t), z_i_(t)), 9 ≤ i ≤ 20. The reference lengths of strides were calculated from the positions of the infrared marker M7 attached to the heel.

**Figure 2:**
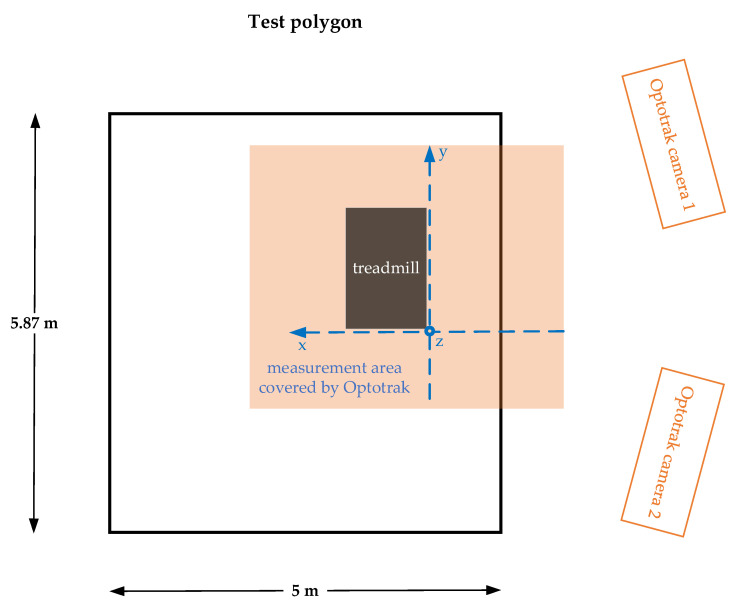
Test polygon for treadmill and rectangular path walking with the defined coordinate system of Optotrak.

The supervisor monitored the experiment to enforce the proper execution according to the following protocol. At first, the treadmill and Optotrak cameras were set up and the experimental area denoted by the yellow tape on the floor. In the experiment, two Optotrak cameras were used at different view angles, thus forming the redundant configuration and minimizing the possibility and duration of the marker’s occlusion. The Optotrak system was calibrated, and cameras aligned according to the selected triaxial cartesian coordinate system as shown in Figure 2. The direction of the *y*-axis was aligned with the direction of walking on the treadmill and with one side of the test polygon. The *z*-axis represented the vertical direction while the *x*-axis represented the direction perpendicular to both the direction of walking and the vertical direction. The experimental area was secured with rope barriers.

**Figure 3:**
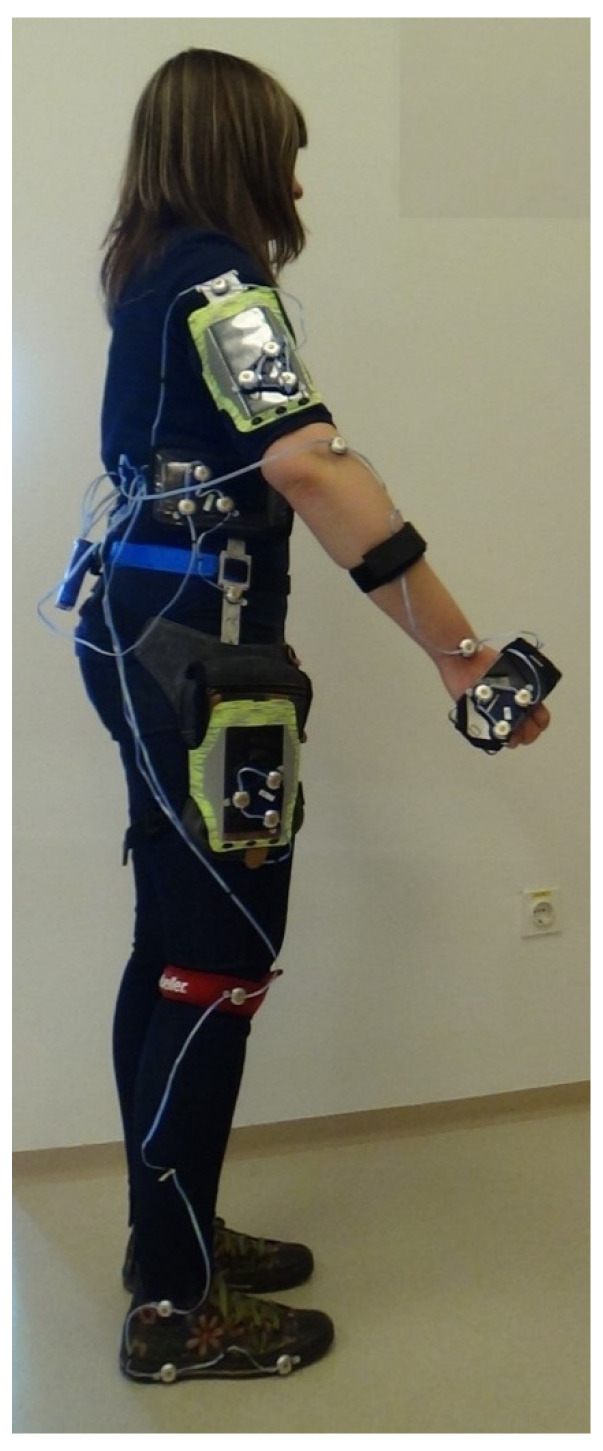
Optotrak’s infrared markers attached to anatomical landmarks of the human body and four smartphones.

Twenty infrared markers were attached to four smartphones and anatomical landmarks on a body as shown in Figure 3. Three infrared markers were attached to anatomical landmarks on the upper extremity (shoulder, elbow, and wrist) and five infrared markers to anatomical landmarks on the lower extremity (between hip and pelvis, knee, ankle, heel, and big toe joint). In addition, three infrared markers for assessing phone position and orientation were attached to the frontal plane of each smartphone. Smartphones’ linear acceleration sensor data were acquired at the maximum sampling frequency. Altogether, 95% of the sampled data were found to be within an inter-sampling interval between 77 and 111 Hz.

To ensure the synchronization of measurements between smartphones and the optical measurement system, a video camera was used for recording the experiments and measuring time. Slow, normal, and fast walking speeds were measured during the first set of experiments. For each walking speed, a person was asked to step onto a treadmill and stand still. The video camera was then turned on and recording began. Next, Optotrak data acquisition was started along with time measurement. Data acquisition on all four smartphones was subsequently enabled. The person was asked to stand still for approximately 10 s, jump in place, and stand still for approximately 10 s afterward. The peaks of the linear acceleration signals recorded by the smartphones, acquired when the heel of the person touched the floor, were used to synchronize the measured signals from the smartphones and the Optotrak system. After the treadmill was turned on and the selected walking speed set, the person walked on the treadmill for a few seconds, so that they could adjust, before starting to count down the time of 15 min. Thereafter, the walking speed on the treadmill was decreased to a stop and the person was asked to stand still so that the data acquisition was manually turned off. Only the preferred walking speed was tested during the second set of experiments aimed for evaluation.

An experimental procedure similar to that used in the first set of experiments was followed for data acquisition in the second set of experiments. The person was asked to stand still at the starting point of the test polygon. After measurement initialization, the person walked 15 min along the marked test polygon with a self-selected preferred constant walking speed. When 15 min elapsed, the person was asked to stop walking anywhere within a few steps after the starting point. The average walking speed of the participants was found to be 4.4 ± 0.6 km/h.

### 2.3. Data Analysis

The data generated were analyzed and processed by using MATLAB (version R2018a 9.4.0.813654), and a clear indication was given when using the MATLAB implementations of functions. Otherwise, the procedures were implemented by authors. The Optotrak markers’ positions were sampled at 100 Hz and pre-processed using a fourth-order lowpass Butterworth filter with a 5 Hz cut-off frequency. The invalid samples due to the short-term marker’s occlusions were prior interpolated with spline interpolation. The MATLAB implementation of the Butterworth filter was used. Smartphone linear acceleration measurements were resampled using linear interpolation to 100 Hz and pre-processed to eliminate errors by employing wavelet denoising, since it has no negative impact on acceleration patterns [57]. The MATLAB implementations of interpolation and wavelet denoising were used. Optotrak’s and smartphone measurements were synchronized with respect to acceleration peaks. The reference positions of the infrared markers attached to the frontal plane of the smartphones were used for aligning the smartphones’ local coordinate systems with the Optotrak’s coordinate system. Of note, a rotation transformation was derived, implemented, and applied for each smartphone to align its coordinate systems to the Optotrak’s coordinate system. On the treadmill, strides were detected by analyzing the displacement of the infrared marker attached to the heel in the walking direction. The MATLAB function findpeaks was used to identify the occurrence of the heel touching the surface of the treadmill, i.e., local maximums. By determining the local minimum within the consecutive detected heel strike events, stride lengths were calculated. Stride detection on smartphones for the treadmill experiments was generated on the smartphones’ outputs by means of the heel strike events determined by the data from the Optotrak’s system. Whereas, on rectangular-shaped test polygon, stride detection was conducted on the smartphones’ outputs by employing the acceleration peak detection algorithm. In a similar way, the MATLAB function findpeaks was used.

### 2.4. Derivation of the Step Length Estimation Model

PCA, a technique that provides enhanced insight into data, was employed in the derivation process of the step length estimation model on the acquired dataset as indicated in Figure 1. The use of PCA reduces the dimensionality of the data, thus limiting the search space, while retaining a large degree of variation from the original dataset [48,49,50]. For each infrared marker attached to the anatomical landmark on the human body, relevant parameters were identified, and their main common characteristics were determined. Next, the correlation between the parameters and stride length was exploited by employing principal component regression. Prior to discussing these steps in more detail, preliminaries that refer to the movement of infrared markers during walking are presented.

#### 2.4.1. Preliminaries

The motion of an infrared marker attached to an anatomical landmark on the human body during walking can be represented with a curve in space [58]. The current position of the infrared marker in space is defined as the vector $\vec{r}$ with three components that represent position coordinates in the *x*-, *y*-, and *z*-direction. These coordinates change with respect to time (t) during walking:

(1) $$\vec{r}=\vec{r}\left(t\right)=\left(x\left(t\right),y\left(t\right),z\left(t\right)\right).$$

The displacement between two consecutive samples $d\vec{r}$ is defined as

(2) $$d\vec{r}=\vec{r}\left(t_{i}\right)-\vec{r}\left(t_{i-1}\right),$$

where $\vec{r}\left(t_{i}\right)$ represents the position of the infrared marker measured at the time $t_{i}$, and $\vec{r}\left(t_{i-1}\right)$ represents the preceding position of the infrared marker measured at the time $t_{i-1}$. For a small enough time difference

(3) $$\;\mathrm{dt}={\;t}_{i}-{\;t}_{i-1},$$

the vector of velocity is defined as:

(4) $$\vec{v}=\frac{d\vec{r}}{\mathrm{dt}}=\dot{r}$$

with the components

(5) $$v_{x}=\frac{\mathrm{dx}}{\mathrm{dt}},\;v_{y}=\frac{\mathrm{dy}}{\mathrm{dt}},\;\mathrm{and}\;v_{z}=\frac{\mathrm{dz}}{\mathrm{dt}}.$$

Vector $\vec{v}$ has the direction of the tangent on the curve in every time instant. The difference between velocities in two consecutive samples is defined as

(6) $$d\vec{v}=\vec{v}\left(t_{i}\right)-\vec{v}\left(t_{i-1}\right).\;$$

For a small enough difference (dt) defined as in (3), the acceleration vector is defined as

(7) $$\vec{a}=\frac{d\vec{v}}{\mathrm{dt}}=\dot{v},$$

with the components

(8) $$a_{x}=\frac{{\mathrm{dv}}_{x}}{\mathrm{dt}},\;a_{y}=\frac{{\mathrm{dv}}_{y}}{\mathrm{dt}},\;\mathrm{and}\;a_{z}=\frac{{\mathrm{dv}}_{z}}{\mathrm{dt}}.$$

The respective velocity and acceleration vectors were calculated for infrared markers by employing finite difference formulas to approximate numerical derivatives, i.e., the central difference for interior data points and single-sided differences for endpoints. All the derivatives were filtered with a moving average filter over 10 data points. The MATLAB implementations of finite difference formulas (gradient function) and moving average filter (movmean function) were used.

#### 2.4.2. PCA and Principal Component Regression

PCA transforms the matrix of input data into principal components—eigenvectors that are essentially orthogonal linear combinations of input parameters—along with eigenvalues that provide information regarding the distortion of the input data [50,59]. The principal component with the highest eigenvalue, therefore, represents the direction where the variance in the dataset is the largest. The main advantage of this technique is reducing the number of interrelated parameters into a small set of representative and uncorrelated parameters while retaining as much of the variability present in the original dataset as possible [49,50].

Firstly, the input data for the PCA were prepared. A matrix $X\in {\mathbb{R}}^{\mathrm{nxp}}$, where *n* represents the overall number of strides and *p* represents the number of potential parameters, was constructed. Each row of matrix *X*, which corresponds to one stride, was filled with the following parameters: the duration of the stride and traveled path in a single stride in *x*-, *y*-, and *z*-directions of the infrared marker. Mean, median, and range values of acceleration and velocity vectors were calculated in *x*-, *y*-, and *z*-directions, as well as for their magnitudes.

Secondly, the PCA was used on the matrix *X*. The MATLAB implementation of the PCA was utilized and yielded the output matrices $Z\in {\mathbb{R}}^{\mathrm{nxp}}$ of principal component scores and $A\in {\mathbb{R}}^{\mathrm{pxp}}$ that represent an orthonormal matrix whose columns are the eigenvectors of the covariance matrix of *X* ordered in the descending order of principal component variances—eigenvalues of the covariance matrix of *X*.

The following relation applies between the principal component scores and the input data:

(9) $$Z=\tilde{X}A,$$

where $\tilde{X}\in \;{\mathbb{R}}^{\mathrm{nxp}}$ is the matrix in which each column mean is subtracted from the corresponding column in the matrix *X* so that the columns total zero means. The derivation of the model is based on the following property [50]. Suppose that $\tilde{X}$ is defined as above and that the corresponding regression equation is:

(10) $$y=\tilde{X}\beta +\epsilon ,$$

where y represents the vector of *n* observations on the dependent variable—stride lengths—measured around the sample mean, β represents the vector of parameters that are yet to be determined in the model, and ϵ represents the error terms. The principal component regression [50] is defined as

(11) y= Zγ+ϵ,

where y, Z, and ϵ are defined as above, and γ is the vector of parameters defined as:

(12) $$\gamma =A^{T}\beta .$$

The least squares estimator for γ is defined as:

(13) $$\hat{\gamma }={\left(Z^{T}Z\right)}^{-1}Z^{T}y,$$

where Z and y are defined as above.

This property implies that the predictor variables in regression analysis could be replaced by their first few principal components [50]. This may not be the best choice to represent the relationship between stride lengths and input parameters, as these values do not meet the criterion set for the new step length estimation model, i.e., the minimum number of input parameters.

Since the matrix A represents an orthonormal basis, principal component regression can be defined in the reduced form also:

(14) $$y={\;Z}_{m}\gamma_{m}+\epsilon_{m},$$

where $\gamma_{m}$ represents a vector of m elements that are a subset of elements of γ, $Z_{m}\in {\mathbb{R}}^{\mathrm{nxm}}$ is a matrix whose columns are the corresponding subset of columns of Z, and $\epsilon_{m}$ is the appropriate error term [50]. The number of input parameters in principal component regression can, therefore, be arbitrary, hence the value of m was chosen to be one so that the proposed model would include the minimum number of input parameters. When choosing the subset elements in (14), one has to eliminate large variances due to multicollinearities. This is accomplished by deleting the principal components whose variance inflation factors are large [50]. A variance inflation factor for the *j*-th variable is defined as the *j*-th diagonal element of ${\left({\tilde{X}}^{T}\tilde{X}\right)}^{-1}$. In the case of uncorrelated variables, the values of variance inflation factors are one.

For each parameter, the variance inflation factors were calculated and the parameters that corresponded to the large values of variance inflation factors were excluded from the study. Then, the absolute values of the sums of error terms $\epsilon_{1}$ were calculated for each remaining parameter, and the parameter that had the minimum value was chosen for use as the basis of the model. The parameter was the range of the acceleration magnitude. The chosen relation between the stride length and the range of acceleration magnitude within the stride is as follows:

(15) $$d_{\mathrm{est}}=a_{\mathrm{range}}^{0.1}\beta +\epsilon ,$$

where $d_{\mathrm{est}}$ represents the estimated stride length, $a_{\mathrm{range}}$ stands for the range of acceleration magnitude, $\beta \in {\mathbb{R}}^{+}$ represents the tunable constant, and ϵ represents the error term. Since the walking of a non-elderly healthy person can be considered symmetrical in terms of both spatial and temporal parameters regardless of gender [54], the proposed model can be used for step length estimation as well.

### 2.5. Evaluation

The proposed model was evaluated on the dataset, which contains the measurement outputs of four off-the-shelf smartphones for different walking speeds for long-term walking on the treadmill and on the polygon. The same subjects participated in these experiments as in those for the derivation of the model. The performance of the proposed model was compared to the performances of the selected models in terms of accuracy of stride length estimation and accuracy of estimated walked distance.

The criteria for the selection of the models used for comparison were based on the presence of an adequate description for the implementation, the basis of the model, and subsequently, the input of the model. As one of the aims of this work was to advance knowledge and gain more insight into acceleration-based models, a number of representative-related acceleration-based models was included in the comparison. In particular, those with the equation for step length estimation similar to the proposed model were selected, so that all models could be tuned in the same way. As a consequence, the models proposed by Weinberg [33], Kim et al. [34], and Zijlstra and Hof [42] were selected for the comparison. In addition, the step-frequency-based model proposed by Tian et al. [21] was included in the comparison as a reference, since it has the same form of the equation for step length estimation as the proposed model and achieved steady performance. Table 1 summarizes the characteristics of the models.

The first part of the evaluation was carried out on the data collected during the experiments on the treadmill where different walking speeds (slow, normal, and fast) were assessed. Since all the models include tunable constants, the first 5 min of the data acquired in each assessment track were used for the tuning and the last 10 min of the data acquired in each assessment track for the performance evaluation. All models were tuned with personalized constants calculated by employing the least squares estimator as shown in (13). Personalized constants calculated for each person per smartphone and assessment track were utilized for the performance evaluation of stride length estimation on the second part of the same assessment track, resulting in 120 personalized constants calculated for each model.

The second part of the evaluation was carried out on the data collected on a rectangular test polygon. Again, all models were tuned with universal constants calculated by merging the first 5 min of the data acquired in each assessment track on the treadmill and employing the least squares estimator as shown in (13). One universal constant was calculated for each model and employed to calculate the walked distance on the polygon.

For the experiments on the treadmill, in which the models were tuned with personalized constants, the performance of the models in terms of the accuracy of stride length estimation was calculated as the absolute difference between the estimated stride length and measured stride length. For the experiments on the test polygon, the performance of the model was calculated as the accuracy of estimated walked distance p as

(16) $$p=\frac{\left|d_{est}-d^{*}\right|}{d^{*}}\cdot 100\%$$

where $d_{est}$ represents the walked distance estimated by the model, and $d^{*}$ the exact walked distance. The accuracy of the estimated walked distance was calculated for the models tuned with universal constants.

## 3. Results

In this section, the results of the evaluation of the proposed model are shown in comparison to the selected models. Firstly, the results of the evaluation on the treadmill are presented, where the stride length estimation errors for each walking speed per sensor position are listed. Next, the results of the evaluation on the rectangular-shaped test polygon are presented, where the path length estimation errors for each of the selected models are listed.

### 3.1. Treadmill Experiment

#### 3.1.1. Overall Results

Mean absolute errors (MAEs) and standard deviations (SDs) for the overall stride length estimation are shown in Table 2. MAEs of stride lengths estimated by the models range from 6.44 to 10.38 cm, and their SDs vary from 4.68 to 8.31 cm. Results indicate that the proposed model estimated stride lengths more accurately than other models selected for the comparison, i.e., approximately 0.5 cm per stride than the model proposed by Weinberg [33] that yielded the second-best results.

**Figure 4:**
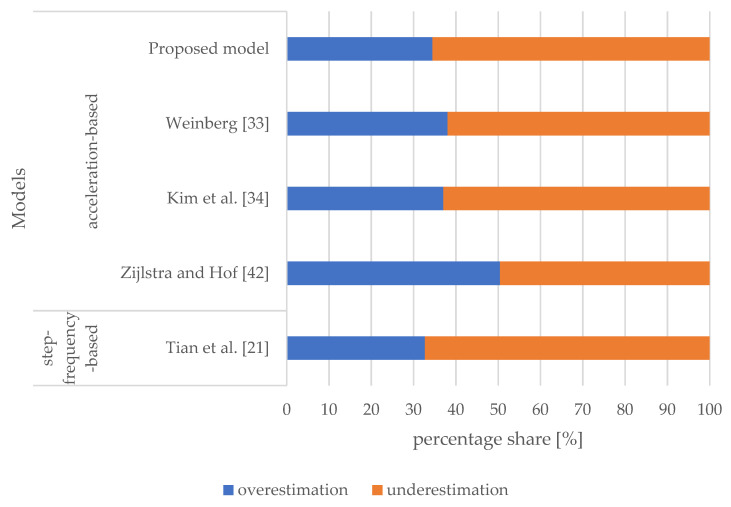
Percentage shares of overestimated and underestimated stride lengths for the selected models.

Figure 4 shows the percentage shares of overestimated and underestimated stride lengths for the selected models. All models investigated generally tend to underestimate the majority of stride lengths, and the percentage share of the proposed model is comparable to the results of the other models selected for comparison.

**Figure 5:**
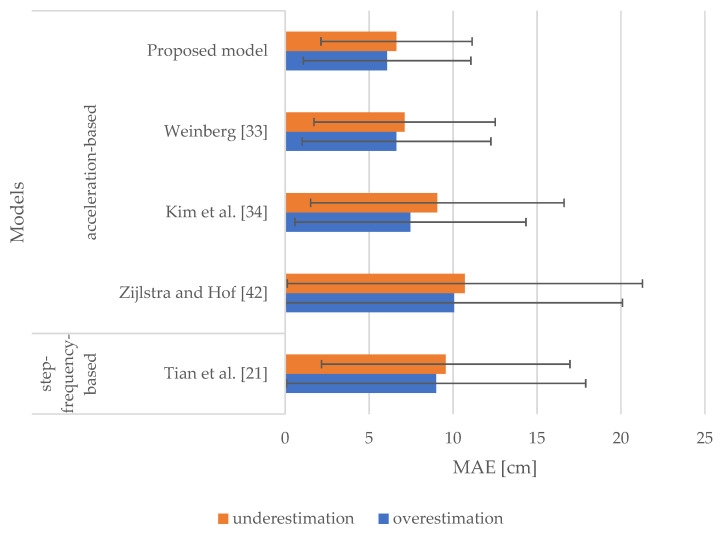
MAEs and SDs of overestimated and underestimated stride lengths for the selected models.

Figure 5 demonstrates MAEs and the corresponding SDs of the overestimated and underestimated stride lengths. MAEs range from 6.07 to 10.70 cm, whereas the corresponding SDs vary from 4.50 to 10.58 cm. Results indicate that the proposed model slightly outperformed all the selected models in terms of accuracy of stride length estimation. It produced an MAE of underestimated stride lengths of 6.63 cm, which is 0.48 cm less than the model proposed by Weinberg [33]. Similarly, the proposed model outperformed all of the other models in terms of overestimated stride lengths. More specifically, it produced an MAE of 6.07 cm, which is 0.56 cm less than the model proposed by Weinberg [33]. This model again yielded the second-best results. The largest difference between the MAEs of the overestimated and underestimated stride lengths was observed with the model proposed by Kim et al. [34], whereas the smallest difference was observed with the model proposed by Weinberg [33]. Their values were found to be 1.60 and 0.48 cm, respectively. The difference between the MAEs of the overestimated and underestimated stride lengths in our proposed model was 0.56 cm, which is the second lowest amongst all the models compared.

Since the proposed model produced comparable results to the selected models in terms of the percentage shares, MAEs, and differences between the overestimated and underestimated stride lengths, only the overall MAEs of estimated stride lengths per sensor position and walking speed are listed going forward.

#### 3.1.2. Smartphone at Upper Arm

**Figure 6:**
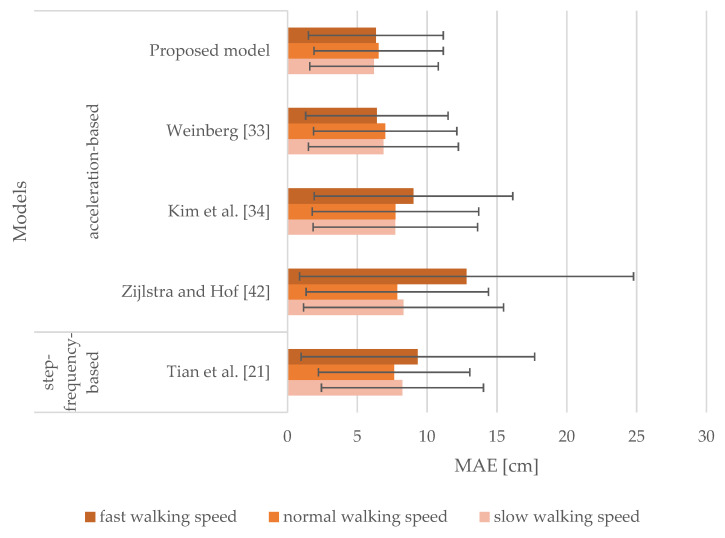
MAEs and SDs of the models for the smartphone attached to the upper arm for slow, normal, and fast walking speeds.

Figure 6 lays out the results achieved from the models where sensor inputs from the smartphone attached to the upper arm were analyzed. It includes MAEs and SDs of stride length estimation for slow, normal, and fast walking speeds. For slow walking speed, MAEs range from 6.20 to 8.31 cm, and the corresponding SDs vary from 4.60 to 7.16 cm. MAEs range from 6.53 to 7.86 cm and from 6.33 to 12.82 cm for normal and fast walking speeds, respectively. Furthermore, the values of SDs are in the range of 4.63 to 6.53 cm for normal walking speed and from 4.83 to 11.96 cm for fast walking speed. The proposed model produced MAEs of 6.20, 6.53, and 6.33 cm for slow, normal, and fast walking speeds, respectively. It was concluded that our proposed model generated the best results, outperforming all the models selected for comparison. These models except the model proposed by Zijlstra and Hof [42] produced MAEs greater by no more than approximately 3 cm for tested walking speeds. The model proposed by Weinberg [33] performed very similarly to the proposed model, yet it produced slightly worse results. The models proposed by Kim et al. [34], Zijlstra and Hof [42], and Tian et al. [21] performed the worst for fast walking speed.

#### 3.1.3. Smartphone at Hand

**Figure 7:**
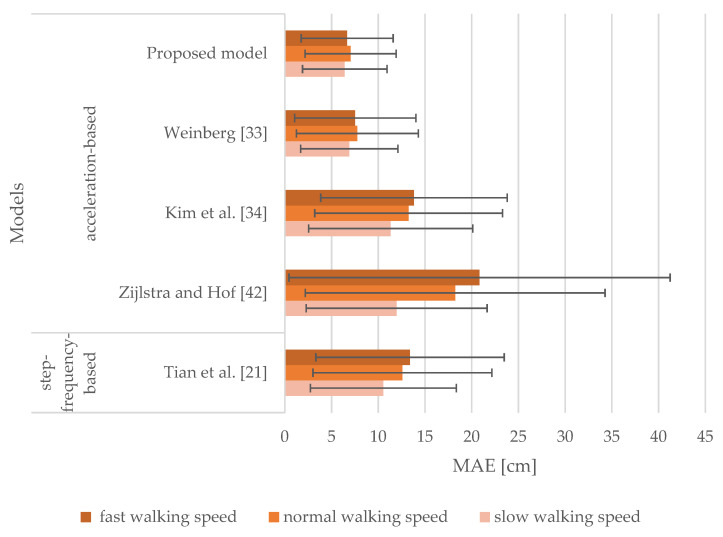
MAEs and SDs of the models for the smartphone attached to the hand for slow, normal, and fast walking speeds.

Figure 7 shows the results of the models when sensor inputs from the smartphone attached to the hand were analyzed. It represents MAEs and SDs of the stride length estimation for slow, normal, and fast walking speeds. MAEs range from 6.41 to 11.97 cm for slow walking speed, whereas for normal and fast walking speeds they range from 7.04 to 18.23 cm and 6.67 to 20.84 cm, respectively. The corresponding SDs range from 4.52 to 9.68 cm, from 4.87 to 16.04 cm, and from 4.92 to 20.40 cm for slow, normal, and fast walking speeds, respectively. The proposed model produced MAEs of 6.41, 7.04, and 6.67 cm for slow, normal, and fast walking speeds, respectively. It outperformed all the models selected for the comparison. The Weinberg model [33] produced the next best results with MAEs observed to be greater by 0.49, 0.72, and 0.86 cm for slow, normal, and fast walking speeds, respectively. The models proposed by Kim et al. [34] and Tian et al. [21] performed very similarly for tested walking speeds. Notably, the model proposed by Zijlstra and Hof [42] performed the worst.

#### 3.1.4. Smartphone at Pelvis

**Figure 8:**
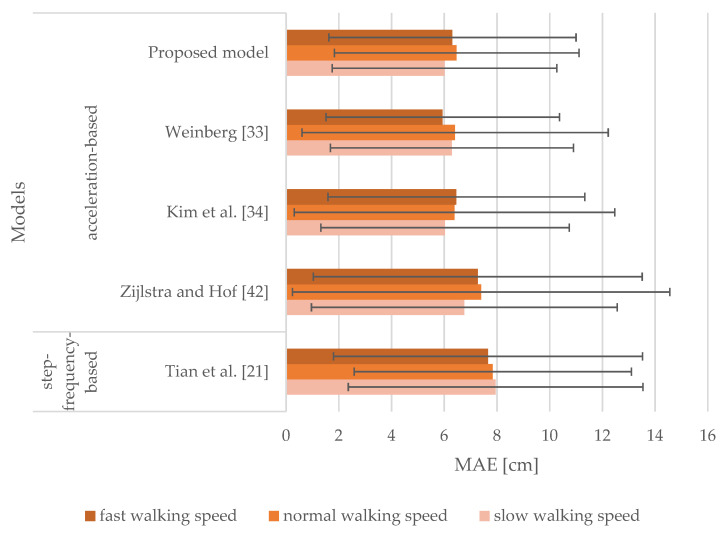
MAEs and SDs of the models for the smartphone attached to the pelvis for slow, normal, and fast walking speeds.

Figure 8 shows the results of the models where sensor inputs from the smartphone attached to the pelvis were investigated. It includes MAEs and SDs of stride length estimation for slow, normal, and fast walking speeds. For slow walking speed, MAEs range from 6.01 to 7.95 cm, and the corresponding SDs vary from 4.26 to 5.80 cm. MAEs range from 6.39 to 7.84 cm and from 5.94 to 7.66 cm for normal and fast walking speeds, respectively. Whereas, the values of SDs are in the ranges of 4.64 to 7.16 cm for normal walking speed and 4.43 to 6.24 cm for fast walking speed. Acceleration-based models performed quite similarly for all walking speeds outperforming the step-frequency-based model proposed by Tian et al. [21]. The proposed model produced MAEs of 6.01, 6.47, and 6.31 cm for slow, normal, and fast walking speeds, respectively. It outperformed all the models selected for comparison for slow walking speed. Whereas, for normal and fast walking speeds, the models proposed by Kim et al. [34] and Weinberg [33] outperformed the proposed model. They produced MAEs of 6.39 and 5.94 cm, respectively. The proposed model yielded performances similar to those observed when attached to the hand.

#### 3.1.5. Smartphone at Thigh

**Figure 9:**
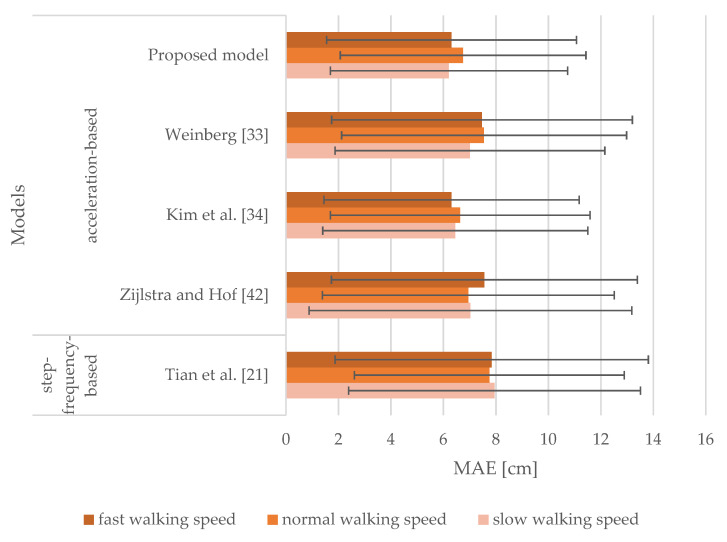
MAEs and SDs of the models for the smartphone attached to the thigh for slow, normal, and fast walking speeds.

Figure 9 shows the results of the models with sensor inputs from the smartphone attached to the thigh. It includes MAEs and SDs of stride length estimation for slow, normal, and fast walking speeds. MAEs range from 6.21 to 7.95 cm for slow walking speed, whereas they range from 6.64 to 7.75 cm and from 6.31 to 7.84 cm for normal and fast walking speeds, respectively. The corresponding SDs range from 4.52 to 6.15 cm, from 4.68 to 5.56 cm, and from 4.76 to 5.97 cm for slow, normal, and fast walking speeds, respectively. Results indicate that all the models performed very similarly. Our proposed model produced MAEs of 6.21, 6.75, and 6.31 cm for slow, normal, and fast walking speeds, respectively. It outperformed all the models selected for the comparison for slow walking speed, whereas the model proposed by Kim et al. [34] outperformed all the models for normal walking speed producing an MAE of 6.64 cm. The proposed model and the model proposed by Kim et al. [34] produced the same results for fast walking speed. The proposed model performed in a comparable manner as when in the hand and at the pelvis.

### 3.2. Evaluation of Walking in the Test Polygon

Table 3 presents results from the evaluation of walking in the test polygon for the selected models when smartphones were attached to the upper arm, hand, pelvis, and thigh. It includes MAEs that range from 4.48 to 21.98% and SDs that range from 2.86 to 14.09%. The rightmost column includes overall MAEs and corresponding SDs of the models for all tested smartphone positions. The proposed model produced an overall MAE of 8.27% outperforming all acceleration-based models selected for the comparison in terms of overall MAEs. This result indicates that our proposed model was least affected by walking speed and smartphone position among acceleration-based models. More specifically, our proposed model performed better when smartphones were attached to the upper arm and hand. The model proposed by Weinberg [33] performed similarly for these smartphone positions. However, it performed worse than our proposed model for the pelvis and thigh positions resulting in an overall MAE of 10.01%. In contrast, the model proposed by Kim et al. [34] performed better for the pelvis and thigh positions than the upper arm and hand positions. It produced an overall MAE of 12.46%, which was quite similar to the overall MAE of the model proposed by Zijlstra and Hof [42]. Notably, the step-frequency-based model proposed by Tian et al. [21] outperformed all the acceleration-based models by producing an overall MAE of 4.75%. This model had the advantage on the test polygon, possibly due to the inclusion of the user’s height.

## 4. Discussion

In this section, the results and findings are discussed starting with the functional comparison of the proposed model with the other models. Results obtained during the evaluation on the treadmill and test polygon are discussed prior to presenting limitations of this study and future research directions.

### 4.1. Functional Comparison

Herein, a new step length estimation model that utilizes acceleration magnitude as the main input has been proposed. It is, therefore, unaffected by smartphone orientation. This offers an important advantage over several models that include acceleration and need properly oriented smartphone placement on the body, e.g., the models proposed by Weinberg [33], Kim et al. [34], and Zijlstra and Hof [42]. The inputs used in these models include acceleration in walking direction (Kim et al. [34]) and vertical acceleration (Weinberg [33] and Zijlstra and Hof [42]). Similar to the proposed model, step-frequency-based models are also unaffected by smartphone position.

Moreover, the proposed model does not include any user-specific parameters, such as the user’s height or leg length, unlike several other models. For example, the model proposed by Tian et al. [21] includes the user’s height, whereas the model proposed by Zijlstra and Hof [42] includes the user’s leg length. Including such parameters in the model requires users to enter them prior to starting the process of step length estimation.

One tunable constant was included in the proposed model. Similar to the proposed model, one tunable constant is also included in all the models selected for the comparison, i.e., the models proposed by Tian et al. [21], Weinberg [33], Kim et al. [34], and Zijlstra and Hof [42]. Moreover, the equation for step length estimation in these models has a similar form, so all models can be tuned in a similar way by utilizing the least squares estimator. The tuning of models that include more than one tunable constant would be more computationally complex when compared to the tuning of the model with one tunable constant. In general, it depends on the placement of the tunable constant in the step length estimation equation.

To sum up, the proposed model calculates step length by utilizing acceleration magnitude as the main input offering an important advantage over compared acceleration-based models that need properly oriented smartphone placement on the body. In addition, it also includes one tunable constant making it less computationally complex to tune when compared to the models that include two or more tunable constants. Moreover, it does not include any user-specific parameters, such as the user’s leg length or height. Due to these characteristics, the proposed model could present an appealing alternative amongst acceleration-based models that could be used for step length estimation.

### 4.2. Treadmill Experiment

#### 4.2.1. Overall Results

Results shown in Table 2 indicate that the proposed model outperformed all the models (acceleration-based and step-frequency-based) in terms of the overall accuracy of stride length estimation by producing an MAE of 6.44 cm. Results presented in Figure 5 indicate that the proposed model performed in a comparable manner in terms of overestimated and underestimated stride lengths. The acceleration-based model proposed by Weinberg [33] performed similar to our proposed model, yet slightly worse. The model proposed by Kim et al. [34] produced a slightly larger error of underestimated stride lengths. All models that include user-specific parameters, i.e., the models proposed by Tian et al. [21] and Zijlstra and Hof [42], performed the worst. All models selected for comparison generally tend to underestimate the majority of stride lengths.

#### 4.2.2. The Impact of Smartphone Position and Walking Speed

The results obtained from Figure 6, Figure 7, Figure 8 and Figure 9 indicate that the walking speed and smartphone position did not affect the performance of the proposed model: the MAEs of stride length estimation ranged from 6.01 to 7.04 cm indicating a steady performance in different circumstances when tuned with personalized constants.

When the smartphone was attached to the upper arm, the proposed model outperformed all the models selected for the comparison regardless of walking speed. Our proposed model performed in a comparable manner when smartphones were attached to the hand, pelvis, and thigh. Notably, the model proposed by Zijlstra and Hof [42] exhibited a less favorable performance for normal and fast walking speeds when attached to the hand or the upper arm. This model was derived by observing the vertical displacement of the center of body mass during walking, thus not emulating the motion of the hand during walking. The model proposed by Tian et al. [21] yielded steady performance for all tested smartphone positions, but it was outperformed by the model proposed by Weinberg [33]. The latter performed in a manner comparable to our proposed model, yet worse on average. The model proposed by Kim et al. [34] also yielded steady performance for all tested smartphone positions, especially when smartphones were attached to the thigh and pelvis. Nevertheless, this model was affected by walking speed for the hand and upper arm positions similar to the models proposed by Tian et al. [21] and Zijlstra and Hof [42]. When smartphones were attached to the pelvis and thigh, the models were mostly unaffected by walking speed, as the MAEs were found to be in the range of 5.94 to 7.95 cm.

### 4.3. Evaluation in the Test Polygon

Overall, the results in Table 3 indicate that the proposed model outperformed all compared acceleration-based models. Furthermore, results also indicate that our proposed model was the least affected by walking speed and smartphone position amongst the acceleration-based models, i.e., the models proposed by Weinberg [33], Kim et al. [34], and Zijlstra and Hof [42], when tuned with one universal constant. Nevertheless, the performance of the proposed model was worse when compared to the step-frequency-based model proposed by Tian et al. [21], which outperformed all the models selected for comparison. This model yielded steady performance for all smartphone positions. It also includes the user’s height, and this information might have given the model an advantage on the test polygon where participants selected the walking speed to their preference.

### 4.4. Limitations and Future Directions

The results of the proposed model are very promising and on par with the previously discussed acceleration-based models making it an appealing alternative that warrants future research. Before presenting future directions, several limitations of this study are discussed.

Firstly, more subjects could be included in the experiments. In addition, these experiments could also include scenarios of typical smartphone users, e.g., the smartphone placed in a pocket or held in the hand so that the user is reading the content on the smartphone’s screen. By forgoing the tracking of smartphones using the optical measuring system, these scenarios could be simulated, and obtained results would reflect the performance of the models under more real-life circumstances. Secondly, including the minimum number of input parameters in the proposed model limited the search space of the model. By allowing more than one input parameter or more than one tunable constant in the model, a more accurate solution could be obtained.

The results indicate that our proposed model outperformed all acceleration-based models selected for the comparison. It also outperformed the step-frequency-based model proposed by Tian et al. [21], but only in the experiments on the treadmill, where it was tuned with personalized constants. One direction for future research would be to investigate the link between the values of tunable constant under different experimental circumstances. The result of this research could be the generation of an algorithm for automatically tuning the model. Another direction for future research could be to extend the proposed model with additional parameters, e.g., the user’s height or step frequency, to improve the performance.

## 5. Conclusions

In this paper, we presented a novel step length estimation model based on acceleration magnitude input. To the best of our knowledge, we were the first to employ PCA for the derivation of the model, which is based on the kinematics of motion of the human body during walking. The proposed model is unaffected by smartphone orientation. This is an important advantage over compared acceleration-based approaches that all need properly oriented smartphone placement at the body.

We evaluated the proposed model at four typical smartphone positions in slow, normal, and fast walking speeds on the treadmill, where we monitored stride length estimation error, and on the rectangular-shaped test path where we monitored the estimated walked distance for self-selected walking speed. Altogether, 10 persons participated in the experiment doing 15-min-long walks. Results indicate that the proposed model outperformed all acceleration-based models selected for comparison. Furthermore, it was least affected by walking speed and smartphone position amongst acceleration-based models.

All the data used for evaluation are openly available in a repository that we have already established [56] to promote the best practices, increase the comparability of evaluation results, and foster collaboration to share and exchange information. All the other researchers are, therefore, kindly invited to use the data and to contribute to the repository.

## Figures and Tables

**Table 1:** Step length estimation models selected for the comparison, their properties, and information about the participants.

Model	Input	Equation	Basis	Number of Subjects	Height of Subjects
Weinberg [33]	Maximum vertical acceleration values within a step *a_max_*, minimum vertical acceleration values within a step *a_mi_*_n_, tunable constant K	$K\cdot \sqrt[{4}]{a_{max}-a_{min}}$	Inverted pendulum model	Not reported	Not reported
Kim et al. [34]	Mean absolute acceleration value in walking direction within a step *a_mean_*, tunable constant K	$K\cdot \sqrt[{3}]{a_{mean}}$	Approximate third root relation of step length with mean acceleration in walking direction within a step	1	1.75 m
Zijlstra and Hof [42]	Vertical pelvis displacement within a step *V* that is calculated using double integration of acceleration, user’s leg length L	$2\cdot K\cdot \sqrt{2\cdot L\cdot V-V^{2}}$	Inverted pendulum model	15 (treadmill walking), 10 (over ground walking)	Not reported
Tian et al. [21]	Step frequency *F*, user’s height *h*, tunable constant *K*	$K\cdot h\cdot \sqrt{F}$	Approximate square root relation of step length with step frequency	10	In the range of 1.56 to 1.83 m

**Table 2:** MAEs and SDs for overall stride length estimation.

Models		MAE [cm]	SD [cm]
Acceleration-based	Proposed model	6.44	4.68
	Weinberg [33]	6.93	5.49
	Kim et al. [34]	8.46	7.37
	Zijlstra and Hof [42]	10.38	7.54
Step-frequency-based	Tian et al. [21]	9.37	8.31

**Table 3:** MAEs and SDs of walked distances estimated by the selected models for smartphones attached to the upper arm, hand, pelvis, and thigh.

Models		Upper Arm		Hand		Pelvis		Thigh		Overall	
		MAE [%]	SD [%]	MAE [%]	SD [%]	MAE [%]	SD [%]	MAE [%]	SD [%]	MAE [%]	SD [%]
Acceleration-based	Proposed model	5.85	4.45	6.83	3.76	8.42	4.44	11.99	5.37	8.27	4.96
	Weinberg [33]	6.84	5.91	5.94	6.31	8.69	5.15	18.58	9.97	10.01	8.51
	Kim et al. [34]	16.86	7.52	19.43	14.09	5.07	4.58	8.47	5.41	12.46	10.29
	Zijlstra and Hof [42]	7.00	3.89	21.98	13.30	11.89	7.07	9.60	6.37	12.62	9.91
Step-frequency-based	Tian et al. [21]	4.70	3.09	5.26	3.66	4.48	2.86	4.54	2.98	4.75	3.05

## Data Availability

All the evaluation data are publicly available at the open repository for evaluation at https://github.com/repositoryadmin/SLERepository.
